# Infectious Complications in Laparoscopic Gynecologic Oncology Surgery within an ERAS-Compliant Setting

**DOI:** 10.3390/jpm14020147

**Published:** 2024-01-29

**Authors:** Vito Andrea Capozzi, Alessandra De Finis, Elisa Scarpelli, Asya Gallinelli, Luciano Monfardini, Stefano Cianci, Ferdinando Antonio Gulino, Isabella Rotondella, Gabriella Maria Celora, Giulia Martignon, Tullio Ghi, Roberto Berretta

**Affiliations:** 1 Department of Medicine and Surgery, University Hospital of Parma, 43125 Parma, Italy; 2 Unit of Gynecology and Obstetric, Department of Human Pathology of Adult and Childhood “G. Barresi”, University of Messina, 98125 Messina, Italy; 3 Unit of Gynecology and Obstetrics, Department of Human Pathology of Adults and Developmental Age, University Hospital “G. Martino”, 98100 Messina, Italy

**Keywords:** minimally invasive surgery (MIS), gynecologic oncology, postoperative complications, enhanced recovery after surgery (ERAS), infectious complications, laparoscopic surgery, morbidity prevention, gynecologic malignancies

## Abstract

Minimally Invasive Surgery (MIS) represents a safe and feasible option for the surgical treatment of gynecologic malignancies, offering benefits, including reduced blood loss, lower complications, and faster recovery, without compromising oncological outcomes in selected patients. MIS is widely accepted in early-stage gynecologic malignancies, including endometrial cancer, cervical tumors measuring 2 cm or less, and early-stage ovarian cancer, considering the risk of surgical spillage. Despite its advantages, MIS does not rule out the possibility of adverse events such as postoperative infections. This retrospective study on 260 patients undergoing laparoscopic surgery at Parma University Hospital for gynecologic malignancies explores the incidence and risk factors of postoperative infectious complications. The Clavien-Dindo classification was used to rank postoperative surgical complications occurring 30 days after surgery and Enhanced Recovery After Surgery (ERAS) recommendations put into practice. In our population, 15 (5.8%) patients developed infectious complications, predominantly urinary tract infections (9, 3.5%). Longer surgical procedures were independently associated with higher postoperative infection risk (*p* = 0.045). Furthermore, C1 radical hysterectomy correlated significantly with infectious complications (*p* = 0.001, OR 3.977, 95% CI 1.370–11.544). In conclusion, compared to prior research, our study reported a lower rate of infectious complications occurrence and highlights the importance of adopting infection prevention measures.

## 1. Introduction

Over the past twenty years, Minimally Invasive Surgery (MIS) has evolved into the gold standard for the surgical management of numerous gynecologic conditions [1]. As extensively demonstrated, minimally invasive approaches to hysterectomy have resulted in reduced intraoperative blood loss, analgesic requirements, perioperative complications, and length of hospitalization, facilitating a quicker return to daily activities compared to open surgery [2].

In the field of gynecologic oncology, MIS has emerged as a reproducible and safe approach under specific circumstances [3]. Due to its technical feasibility and comparable oncological outcomes to laparotomy, laparoscopy is recommended as the elective treatment for endometrial cancer (EC). Furthermore, MIS has proven its advantages in obese patients with EC, allowing economic savings and reducing post-operative complications [4].

Concerning the surgical treatment of cervical cancer (CC), in 2018, the LACC trial demonstrated that minimally invasive radical hysterectomy was associated with a higher rate of recurrence and lower rates of disease-free survival and overall survival compared to open surgery [5]. Consequently, open abdominal radical hysterectomy has become the recommended surgical approach for women with cervical cancer eligible for surgical treatment. However, the impressive and unexpected results of the LACC trial have led to further investigations into the role of MIS in the treatment of CC. The international multicenter SUCCOR study, in particular, provided retrospective evidence supporting the safety of MIS in stage IB1 cervical tumors with sizes up to 2 cm [6]. This has led to a renewed interest in MIS in the treatment of early-stage CC, although with careful selection criteria, including tumor size and surgeon expertise [7]. In summary, despite the ongoing debate, current evidence suggests that MIS can be safely employed for low-volume tumors.

The latest guidelines from ESGO/ESTRO/ESP also indicate that laparoscopy may be considered for tumors smaller than 2 cm with free margins after conization. However, this procedure should be conducted by highly experienced surgeons [8]. In light of these results, recently within the SHAPE trial, a randomized trial comparing oncological outcomes of simple hysterectomy vs. radical hysterectomy, 75% of patients underwent MIS (50% laparoscopic, 25% robotic) hysterectomy [9]. Furthermore, following the advantages offered by robotic surgery in terms of better maneuverability and visualization, the non-inferiority of robotic radical hysterectomy compared to open surgery has been hypothesized and is currently being investigated in the RACC trial, encompassing all early-stage tumors, including those larger than 2 cm [10].

In the context of ovarian tumors, a distinction is necessary between borderline tumors and invasive tumors. Since borderline ovarian tumors exhibit a higher prevalence in young women and are generally diagnosed at an early stage, laparoscopy is generally preferred as it is associated with more favourable surgical outcomes, including reduced operative time and blood loss, as well as shorter hospital stays, while maintaining similar oncologic outcomes [11,12]. Moreover, minimally invasive approaches play a key role in fertility-sparing surgery (FSS), reducing postoperative complications and preserving childbearing desire [13].

Similar observations can be applied to early-stage OC, with no evidence of extra-ovarian disease. In this case, MIS can be used, but it is mandatory to avoid surgical spillage, making tumor size and surgeon experience crucial [14,15], although strong evidence from the literature is currently lacking.

Concerning advanced-stage ovarian cancer (OC), the role of MIS is still to be clarified. While international guidelines recommend laparotomy as the preferred surgical approach, laparoscopy has now taken over as the prevailing method for resectability assessment in advanced-stage OC, avoiding exploratory laparotomies [16]. Moreover, the laparoscopic approach is under investigation for interval debulking surgery in randomized clinical trials for OC patients undergoing neoadjuvant chemotherapy [17,18].

When dealing with mesenchymal malignancies of the uterus, minimally invasive surgery (MIS) is not recommended. Open laparotomy is preferred as it poses minimal risk for surgical tumor rupture and spillage, which could impact prognosis [19]. However, these types of tumors are often identified only at the final pathology stage. It is estimated that less than 1% of patients undergoing hysterectomy for fibroids will be diagnosed with sarcoma, and only a quarter of patients suspected to have sarcoma based on preoperative findings have the condition [20].

Consequently, there is a risk that patients, initially diagnosed with a large leiomyoma, may undergo MIS without the awareness of an underlying mesenchymal malignancy [21].

Despite the numerous advantages, MIS does not eliminate the possibility of adverse events. Nevertheless, the assessment of the impact of concomitant gynecologic malignancies on surgical morbidity lacks uniformity. In a retrospective analysis involving 1649 women undergoing hysterectomy for benign and malignant indications, Lee et al. reported that malignancy and prior open abdominal surgery emerged as significant risk factors for 30-day readmission, underscoring the importance of identifying high-risk patients during the hysterectomy process for targeted interventions [22]. In a meta-analysis conducted by Radosa, involving ten case series and 7438 patients undergoing laparoscopic surgery for both benign and malignant conditions, the presence of malignant pathology did not prove to be an independent risk factor for the occurrence of complications. The factor most strongly implicated turned out to be the surgeon’s skill [23].

When considering laparoscopy for gynecologic conditions in general, the literature reports a complication rate ranging from 0.2% to 18.0% [2,23]. Furthermore, MIS-related deaths have an incidence of 0.02% and are predominantly due to major retroperitoneal vessel injuries, or less frequently to bowel perforation and subsequent sepsis [24]. In this context, infectious complications can also be a potential cause of life-threatening events, such as severe sepsis and the need for second surgery. In milder cases, they are a preventable cause of post-operative morbidity and prolonged hospitalization [25].

However, there is limited data on the incidence of postoperative infections after laparoscopic surgery for gynecologic malignancies. Regarding total hysterectomy performance, infectious complications account for less than 10.0% of cases independently from the surgical approach, and 9.0% of cases when considering the laparoscopic approach [26]. Postoperative infections in gynecologic surgery often appear as polymicrobial entities due to the association of ascending microorganisms from the vagina and bacterial skin flora [26]. The spectrum of these infections includes vaginal cuff cellulitis, infected hematoma or abscess, surgical site infection, urinary tract infections (UTIs), and respiratory infections [27]. Risk factors contributing to the development of postoperative infectious complications encompass uncontrolled diabetes, smoking, obesity, ineffective host defense, and prolonged hospital stays [27]. Among these, surgical site infection (SSI) stands out as the most common entity associated with pelvic surgery [28]. To mitigate the risk of SSI, the implementation of aseptic techniques and antibiotic prophylaxis is considered crucial. The choice of chlorhexidine-alcohol for surgical site skin preparation has gained widespread acceptance, proving to be superior to povidone-iodine. Additionally, perioperative antimicrobial prophylaxis with cephalosporins is the recommended approach in gynecologic oncology [29,30]. However, despite these established practices, there is currently a lack of studies investigating the occurrence of infectious complications and predisposing conditions in patients undergoing surgery for gynecologic malignancies.

This study aims to bridge this gap, focusing on the evaluation of the incidence and risk factors associated with postoperative infectious complications after laparoscopic surgery in gynecologic oncology.

## 2. Materials and Methods

A retrospective cohort analysis was carried out, involving individuals who consecutively underwent laparoscopic surgery for gynecologic malignancies at the Department of Medicine and Surgery, University Hospital of Parma, spanning from January 2017 to December 2021. The inclusion criteria comprised women affected by endometrial cancer (EC), ovarian cancer (OC), cervical cancer (CC), uterine sarcoma, and borderline ovarian tumors (BOT). Data collection included patients’ characteristics, the American Society of Anesthesiologists (ASA) status, the International Federation of Gynecology and Obstetrics (FIGO) stage, and postoperative infectious complications along with their management. The specific types of complications reported included urinary tract infections (UTIs), abdominal abscesses, surgical site infections, and respiratory tract infections.

Exclusion criteria for the analysis involved incomplete clinical data, the use of surgical approaches other than laparoscopy, and surgeries conducted for benign conditions. Notably, all surgical procedures were performed by expert gynecologic oncology surgeons. Diagnosis of complications was carried out through blood tests, clinical examinations, and radiological assessments. The Clavien-Dindo classification, a widely recognized system for ranking postoperative surgical complications, was employed to categorize complications occurring 30 days after surgery into five severity grades [31]. Following ERAS recommendations [29,30], the institution implemented surgical site infection reduction bundles, encompassing practices such as checking blood glucose levels to reduce perioperative hyperglycemia, administering antibiotic prophylaxis (2 g single-dose cefazolin) to all patients one hour before surgery, and performing skin preparation with a chlorhexidine-alcohol solution. Other measures included placing a urinary catheter before surgery initiation, removing it one day after surgery, utilizing a forced air blanket device to prevent intraoperative hypothermia, and tailoring drain placement based on the surgical procedure. Drain placement was typically restricted to extended lymphadenectomy, bowel resection, or intraoperative complications such as urinary or bowel injuries or significant bleeding. When inserted, the drain is generally left in place until mobilization and/or restoration of bowel function. Postoperatively, patients were managed with vital signs monitoring, blood count control, and daily wound care. Early mobilization on the first day after surgery was encouraged, according to ERAS protocols. Primary indicators of potential infection included fever, tachycardia, and an elevated white blood cell (WBC) count. In cases of multiple fever spikes, urine and blood cultures were obtained, antibiotic therapy was initiated, and radiological examinations were conducted based on clinical presentation. Before discharge, a gynecological examination was performed, and patients were advised to seek emergency care in case of any deviations from the normal postoperative course.

The study received approval from the Parma Ethics Committee under code 23/2022/OSS/AOUPR.

### Statistical Analysis

Quantitative variables were expressed as numbers, percentages, median, and range. Multinomial logistic regression was used to evaluate the correlation between the categorical variables analyzed and the onset of infectious complications. In these cases, the *T*-test and Chi-square test were used to establish statistical significance (*p* < 0.05). Binary logistic regression analysis was used to model the relationships between independent variables (age, BMI, estimated blood loss, and operation time) and the categorical dependent variable (infectious complications). Statistical analysis was performed using IBM SPSS Statistics version 28.0.

## 3. Results

Two hundred and sixty total patients met the inclusion criteria. One-hundred eighty patients affected by EC (69.2%), 18 cases of CC (6.9%), 51 patients with BOT (19.6%), 9 cases of OC (3.5%), and 2 patients with uterine sarcoma (0.8%), were included in the study. All patients underwent laparoscopic surgical treatment for primary disease. In the case of patients with uterine sarcoma, the diagnosis was performed incidentally, in the absence of preoperative suspicion for malignancy. The details of the surgical interventions and patients’ characteristics are shown in Table 1 and Table 2. Of the entire series, 15 (5.8%) total infectious complications were recorded (Table 3): 9 cases of UTIs (3.5%), 1 case of pneumonia (0.4%), 3 patients with an abdominal abscess (1.15%), 1 vaginal vault infection (0.4%), and 1 skin scar infection (0.4%). The multinomial logistic regression is reported in Table 4. Of the variables analyzed, only C1 radical hysterectomy was significantly associated with the occurrence of infectious complications (*p* = 0.001, Odds Ratio 3.977, 95% Confidence Interval 1.370–11.544). Of the 51 patients who underwent a radical hysterectomy, 7 (13.7%) cases developed infectious complications: 5 UTIs, 1 vaginal vault infection, and 1 abdominal abscess. The median duration of surgery was 110 min (40–407 min). The operation time (see Table 5) was an independent factor statistically correlated to infectious complications (*p* = 0.045). The Binary Logistic regression analysis showed that with every minute of surgery, the risk of infectious complications increased 1.009 times (95% CI 1.000–1.017).

## 4. Discussion

### 4.1. Results in the Context of Published Literature

In the last few years, there has been an increasing trend in MIS utilization for gynecologic malignancies, with reassuring evidence on oncological outcomes and low rate of postoperative complications [1,4,6,17,18]. Independently from the surgical approach, the most common postoperative infections after pelvic surgery include vaginal cuff cellulitis (about 8%), infected hematoma or abscess (about 14%), SSI (about 22%), UTIs (about 13%), and pneumonia (about 2%) [27,32]. In line with these observations, the majority of infections in our population were UTIs, followed by abdominal abscesses and SSI. To the authors’ knowledge, few previous data are available concerning infectious complications after gynecologic oncologic surgery via laparoscopy [23].

In our study, 5.6% of women developed an infectious complication, a lower rate than previously reported after MIS in gynecology (about 10.0%) [2,26]. The differences between our findings and others may be explained by strict adherence to infection prevention measures, according to ERAS protocols [30,33].

In particular, Dedden et al. in a retrospective analysis of patients undergoing a laparoscopic hysterectomy reported an incidence of urinary tract infections (UTIs) of 11.3% after immediate catheter removal and 20.8% after delayed removal [34]. We reported a lower incidence, and the most obvious explanation is the early removal of the urinary catheter (1 day after surgery). Furthermore, they diagnosed UTIs only with analysis of a urine sample looking for leucocytes, erythrocytes, and bacteria, while we considered a positive culture (>105 bacteria/mL) in patients with suspicious symptoms. 

Concerning risk factors for infectious complications, one noteworthy finding from our study was that the duration of surgery is an independent risk factor for postoperative infections. This observation aligns with previous research. Catanzarite et al. analyzed 7630 patients undergoing MIS for gynecologic diseases and found an association between longer operative times and increased overall complication rate, surgical and medical complications [35].

Despite the well-documented association between operative time and complications following laparoscopic hysterectomy for benign diseases, limited data are available in gynecologic oncology. Singh et al. investigated the impact of surgery duration on postoperative morbidity in women undergoing MIS for endometrial cancer. In line with our results, the authors showed that the overall complications rate was almost doubled when surgery lasted more than 240 min. More specifically, the rate of UTIs was 1.8% in patients with operative times < 240 min, vs. 3.2% after 240 min [36].

The complexity of surgical procedures undoubtedly increases the risk of morbidity among patients [37]. Concerning our cohort of women, C1 radical hysterectomy showed a significant association with the occurrence of infectious complications.

Regarding SSI, it is the most common complication associated with gynecologic surgery [28], and operative duration is often cited as an independent and potentially modifiable risk factor for SSI [38]. Intuitively, prolonged operative time and exposition of the surgical site to the environment and manipulation, increase the risk of bacterial contamination. In this study, we report a single case of SSI and the duration of the procedure was among the shortest (90 min) of the entire series. However, the patient’s BMI was 46 kg/m^2^ and this factor highlights the association between obesity and the development of SSI. A retrospective study conducted in 2011 involving gynecologic cancer patients revealed that individuals classified as morbidly obese (BMI > 40) exhibited a tenfold increased risk of wound complications, including surgical site infections (SSI), in comparison to patients within the normal weight range [39].

On the contrary, a systematic review and meta-analysis including 50 studies and 176,016 patients who underwent laparoscopic hysterectomy found no variation in the overall rate of infectious complications based on BMI, aligning with our results [40].

In a large retrospective analysis by Chi et al. increasing age was the most important predictor of complications of laparoscopic surgery performed by a gynecologic oncology service [41]. In contrast to the available evidence, increasing age and blood loss were not significantly associated with the development of postoperative infectious complications in our cohort of women.

### 4.2. Strengths and Weaknesses

One distinctive feature of this study is its specific patient cohort, which comprises women affected by gynecologic malignancies. While other studies primarily focus on benign gynecologic pathologies, our research aims to contribute to the enhancement of postoperative outcomes in this patient subset. It is important to note that all surgical procedures were carried out by highly qualified gynecologic oncology surgeons with at least ten years of surgical experience. Furthermore, the well-recognized Clavien-Dindo Classification system to assess the severity of postoperative complications was used, ensuring a standardized approach to ranking surgical morbidity [31]. However, a notable limitation of this study is its retrospective design, which may have impacted the value of the clinical data.

### 4.3. Implications for Practice

The observed low incidence of postoperative infectious complications in our case series provides further support for the effectiveness of implementing preventive measures to enhance postoperative recovery in gynecologic oncology following MIS. Specifically, it is crucial to incorporate risk-reduction strategies in line with the ERAS recommendations [29,30]. These strategies encompass the administration of appropriate antibiotic prophylaxis, thorough skin preparation with chlorhexidine, prevention of hypothermia, avoidance of drain placement, and the use of urinary catheters for less than 24 h after surgery. Additionally, considering the impact of surgery duration, surgeons should optimize surgical techniques to minimize operative time without compromising patient outcomes. This involves effective teamwork, comprehensive pre-operative planning, the involvement of highly skilled surgeons, and the use of advanced surgical technologies.

**Figure 1:**
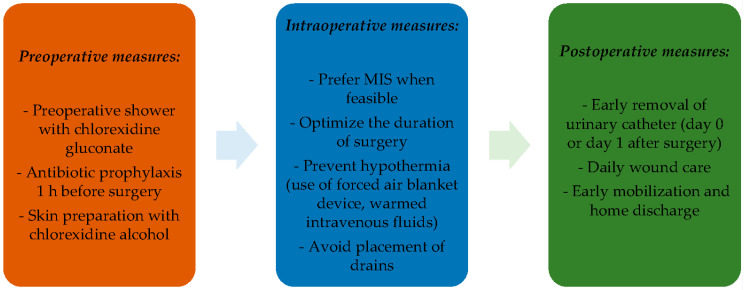
Proposal for a perioperative care bundle for the containment of infectious complications.

The notably low rate of infectious complications in our patient population, compared to previous literature data, underscores the positive impact of these interventions and the expertise of our surgical team on patient outcomes. Based on these encouraging results, in Figure 1 we propose a perioperative care algorithm based on ERAS recommendations and our experience.

## 5. Conclusions

In conclusion, our findings revealed a lower rate of infectious complications (5.8%) compared to existing literature, highlighting the positive impact of stringent infection prevention measures implemented through ERAS protocols.

The analysis underscored the correlation between surgical duration and infectious complications, with C1 radical hysterectomy emerging as a significant contributor. The identification of surgery duration as an independent risk factor emphasizes the necessity for comprehensive investigations into specific elements influencing surgical complications. Our study supports the proposal of a perioperative care algorithm, aligning with ERAS recommendations and our experience, to further optimize patient care in gynecologic oncologic surgery. These insights advance our understanding of MIS outcomes in gynecologic malignancies, emphasizing the effectiveness of preventive interventions. The proposed perioperative care algorithm, detailed in Figure 1, integrates evidence-based practices to guide future endeavors in enhancing patient outcomes in gynecologic oncologic surgery. Our study sets the stage for continued advancements and refinements in preventive strategies within this specialized surgical domain.

## Figures and Tables

**Table 1:** Health history of the patients’.

	Total	Complication *n*; %	No Complication *n*; %
	260; 100	15; 5.8	245; 94.2
Parity	214	14; 93.3	200; 81.6
Age median (years)	63	60	63
BMI median (kg/m^2^)	27	28	26
High blood pressure	94	7; 46.7	87; 35.5
Diabetes	20	1; 6.7	19; 7.8
Hypothyroidism	32	4; 26.7	28; 11.4
Cardiovascular disease	27	3; 20	24; 9.8
Psychiatric disorders	5	1; 6.7	4; 1.6
ASA Status			
1	12	0;	12; 4.9
2	204	10; 66.7	194; 79.2
3	44	5; 33.3	39; 15.9

BMI: body mass index. ASA: American Society of Anesthesiologists.

**Table 2:** Pathological data and surgical procedures performed.

	Total *n*; %	Complication *n*; %	No Complication *n*; %
	260; 100	15; 5.8%	245; 94.2%
Primary disease			
Endometrial Cancer	180; 69.2	11; 73.3	169; 68.9
Cervical Cancer	18; 6.9	1; 6.7	17; 6.9
Ovarian Cancer	9; 3.5	3; 20	6; 2.4
BOT	51; 19.6	0	51; 20.8
Uterine sarcoma	2; 0.8	0	2; 0.8
FIGO stage			
Endometrial Cancer (2009)			
IA	110; 42.3	7; 46.8	103; 42
IB	34; 13.1	0	34; 13.9
II	3; 1.2	1; 6.6	2; 0.8
IIIA	7; 2.7	0	7; 2.9
IIIB	1; 0.4	0	1; 0.4
IIIC	24; 9.2	3; 20	21; 9.3
IVB	1; 0.4	0	1; 0.4
Cervical Cancer (2018)			
IA1	6; 2.3	0	6; 2.5
IA2	2; 0.8	1; 6.6	1; 0.4
IB1	4; 1.5	0	4; 1.5
IB2	3; 1.3	0	3; 1.2
IB3	1; 0.4	0	1; 0.4
IIB	1; 0.4	0	1; 0.4
IIIC	1; 0.4	0	1; 0.4
Ovarian Tumor (2013)			
IA	45; 17.3	2; 13.4	43; 17.5
IB	2; 0.8	0	2; 0.8
IC	5; 1.9	0	5; 2.0
IIA	2; 0.8	0	2; 0.8
IIB	1; 0.4	0	1; 0.4
IIIA	1; 0.4	0	1; 0.4
IIIB	1; 0.4	0	1; 0.4
IIIC	2; 0.8	1; 6.6	1; 0.4
Uterine Sarcoma (2017)			
IB	2; 0.8	0	2; 0.8
LVSI	37; 14.2	4; 26.7	33; 13.5
Grading			
G1	143; 55.0	6; 40	137; 55.9
G2	63; 24.2	6; 40	57; 23.3
G3	54; 20.8	3; 20	51; 20.8
Simple Hysterectomy	82; 31.5	6; 40	76; 31
SLN	115; 44.2	6; 40	109; 44.5
Pelvic LND	58; 22.3	5; 33.3	53; 21.6
Aortic LND	38; 14.6	3; 20	35; 14.3
C1 Hysterectomy	51; 19.6	7; 46.7	44; 17.9
Adjuvant treatment	87; 33.5	5; 33.3	82; 33.5

BOT: borderline ovarian tumor. FIGO: International Federation of Gynaecology and Obstetrics. LND: Lymphadenectomy. LVSI: lymphovascular space invasion. SLN: sentinel lymph node.

**Table 3:** Type of infectious complication and treatment.

Type	Total *n*	Total %	Treatment	Clavien-Dindo
UTIs	9	3.5	Antibiotic therapy	II
Pneumonia	1	0.4	Antibiotic therapy	II
Infected abdominal hematoma	3	1.15	Antibiotic therapy	II
Infected subfascial hematoma			Antibiotic therapy	II
Infected pelvic lymphocele			Antibiotic therapy Surgical decontamination followed by post-operative intensive care admission.	IV
Vaginal cuff abscess	1	0.4	Surgical decontamination of the abscess	IIIB
Skin scar infection	1	0.4	Surgical decontamination	IIIA

UTIs Urinary tract infections.

**Table 4:** Multinomial logistic regression analysis.

	Total	Infectious Complications			
	*n*; %	Yes *n*; %	No *n*; %	*p*-Value	OR (Confidence Interval 95%)
	260	15; 5.8	245; 94.2	-	-
Previous pregnancy	203; 78.1	14; 6.9	189; 93.1	0.436	-
Hypertension	93; 35.8	7; 7.5	86; 92.5	0.521	-
Diabetes	20; 7.7	1; 5	19; 95	0.843	-
Hypothyroidism	32; 12.3	4; 12.5	28; 87.5	0.526	-
Cardiovascular disease	27; 10.4	3; 11.1	24; 88.9	0.787	-
ASA Status				0.144	-
1 2 3	12; 4.6 204; 78.5 44; 16.9	0; - 10; 4.9 5; 11.4	12; 194; 95.1 39; 88.6	- - -	
Previous cesarean section	30; 11.5	3; 10	27; 90	0.216	-
Radical Hysterectomy	51; 19.6	7; 13.7	44; 86.3	0.010	OR 3.977 (95%CI 1.370–11.544)
Sentinel lymph nodes	114; 43.8	6; 5.3	108; 94.7	0.926	-
Pelvic lymphadenectomy	58; 22.3	5; 8.6	53; 91.4	0.231	-
Aortic lymphadenectomy	38; 14.6	3; 7.9	35; 92.1	0.894	-
Bowel Resection	1; 0.4	1; 100	0; 0	0.014	OR 1.071 (95% CI 0.936–1.227)
Appendicectomy	19; 7.3	2; 10.5	17; 89.5	0.405	-
Postoperative Intensive Care Unit	10; 3.8	1; 10	9; 90	0.761	-
Intraoperative blood transfusion	1; 0.4	0 0	1; 100	0.736	-

ASA: American Society of Anesthesiologists. OR: odds ratio.

**Table 5:** Binary Logistic regression analysis between infectious complication occurrence and variables in the column.

	Median (Range)	OR	95% CI	*p*-Value
Age (years)	63 (20–89)	0.983	0.941–1.028	0.458
BMI (kg/m^2^)	27 (18–46)	1.064	0.978–1.158	0.149
Operation time (min)	110 (40–407)	1.009	1.000–1.017	0.045
EBL (mL)	70 (50–1000)	1.002	0.999–1.006	0.246

BMI: Body mass index. EBL: estimated blood loss.

## Data Availability

Data will be provided by the corresponding author upon reasonable request.
